# Fungal Infections Associated with Sphingosine 1-Phosphate Receptor Modulators: Immunological Mechanisms, Clinical Patterns, and Management Considerations

**DOI:** 10.3390/microorganisms14081861

**Published:** 2026-08-21

**Authors:** Kian Michael Yazdan, Sai Tapasvi Madam, Shirisha Pasula

**Affiliations:** 1 College of Osteopathic Medicine of the Pacific, Western University of Health Sciences, Pomona, CA 91766, USA; kian.yazdan@westernu.edu; 2 Division of Infectious Diseases, Department of Medicine, University of California San Francisco, Fresno, CA 93701, USA

**Keywords:** fingolimod, siponimod, sphingosine-1 phosphate receptor modulator, invasive fungal infections, cryptococcal infections, *Cryptococcus*, histoplasmosis, coccidioidomycosis, multiple sclerosis

## Abstract

Fungal infections constitute a large portion of serious infections worldwide and are increasing, partially due to new immunomodulatory therapies, such as Sphingosine-1-Phosphate (S1P) receptor modulators, including fingolimod for the treatment of multiple sclerosis (MS). Fingolimod antagonizes multiple S1P receptors, including S1PR_1_, S1PR_3_, S1PR_4_, and S1PR_5_, resulting in immunological alterations that may increase susceptibility to invasive fungal infections (IFIs). Our PubMed-based literature search identified 43 published cases and a 60-patient case series describing invasive fungal infections in patients receiving S1P receptor modulators. Of these, cryptococcosis was the most frequently documented infection, followed by histoplasmosis and coccidioidomycosis. Most published cases involved patients on fingolimod, whereas evidence for the other medications in this class is extremely limited. Currently, there are no standardized fungal screening protocols. A baseline complete blood count with differentials should be obtained before the initiation of S1P receptor modulator therapy. Patients should also be monitored clinically for signs and symptoms of fungal infection. Primary antifungal prophylaxis and routine serum cryptococcal antigen testing are not currently recommended. Management of suspected IFIs should focus on diagnostic workup to identify the causative organism and extent of organ involvement to determine the appropriate antifungal therapy. Decisions regarding interruption or discontinuation of MS therapy should be individualized according to the clinical syndrome, infection severity, and risk of MS rebound. This narrative review synthesizes the available mechanistic and clinical evidence on invasive fungal infections in patients with multiple sclerosis receiving S1P receptor modulator therapy.

## 1. Introduction

Invasive fungal infections (IFIs) represent a significant yet underrecognized threat to global health, accounting for an estimated 6.5 million cases each year [1,2]. Surveillance data from the United States indicate a mean incidence of 27.2 cases per 100,000 patients per year between 2006 and 2015, a level that has remained consistently high despite advances in antifungal therapy and prophylaxis over the past two decades [3,4]. The sustained burden of fungal infections is largely attributable to an expanded immunocompromised population, including patients with HIV/AIDS, hematologic malignancies, solid organ or hematological transplantation, and those receiving corticosteroids, monoclonal antibodies, biologic therapies, and immunomodulator therapies for autoimmune diseases [5].

Multiple Sclerosis (MS) is a chronic autoimmune demyelinating disorder of the central nervous system (CNS), defined by autoreactive T and B lymphocytes that breach the blood–brain barrier, triggering focal inflammatory lesions, demyelination, and progressive neurological disability [6]. Disease-modifying therapies (DMTs) target this dysregulated immune response through diverse mechanisms, including reducing lymphocyte activation, migration, and survival, thereby limiting CNS inflammation [6]. The DMT landscape in MS is broad, encompassing agents such as interferon-β, glatiramer acetate, teriflunomide, cladribine, dimethyl fumarate, natalizumab, and alemtuzumab, each acting on distinct immunological pathways [7]. Among these, sphingosine-1-phosphate (S1P) receptor modulators represent a mechanistically distinctive class, acting by sequestering lymphocytes within secondary lymphoid organs and preventing their egress into circulation and CNS. Fingolimod (Gilenya) was the first agent in this class and the first oral DMT to receive FDA approval in 2010 for relapsing forms of MS, representing a landmark shift in MS therapeutics [8,9]. Subsequent S1P receptor modulators with greater receptor subtype selectivity have since been approved, including siponimod, ozanimod, and ponesimod, between 2019 and 2021 for relapsing and active progressive forms of MS [8,9]. Beyond MS, S1P receptor modulators are also being utilized or investigated for other inflammatory and immune-mediated conditions, including inflammatory bowel disease, chronic kidney disease, and pancreatic disorders [9,10,11,12,13,14,15].

The growing use of S1P receptor modulators across various indications has brought renewed attention to their infectious sequelae. Although invasive fungal infections are uncommon with the use of S1P receptor modulators, an increasing number of reports have described infections caused by endemic and opportunistic fungi, most notably *Cryptococcus*, *Histoplasma*, and *Coccidioides.* These infections can result in substantial mortality and morbidity, especially when diagnosis is delayed or immunological dysfunction is not recognized. This review discusses the immunological mechanisms of S1P receptor modulators and their association with fungal infection susceptibility, summarizes reported cases of fungal infections, and outlines the key diagnostic and management considerations relevant to the affected population.

## 2. S1P Receptor Modulator Mechanism of Immunosuppression

Sphingolipids are essential structural lipid components of eukaryotic cell membranes and are expressed ubiquitously throughout the body, including the brain parenchyma and lymphocytes [16,17,18]. These lipids are involved in many critical cellular processes, such as proliferation, survival, migration, and regulation of the activation and function of immune cells such as lymphocytes and macrophages through conversion to the bioactive metabolite, sphingosine-1-phosphate (S1P), by sphingosine kinases in response to cytokines, immunoglobulins, hormones, and trophic factors [16,17,18]. S1P then functions as a second messenger, signaling effects through five G protein-coupled receptor pathways [16,17,18,19]. Among S1P receptors (S1PR_1–5_), S1PR_1_ is expressed on most B and T lymphocytes and plays a central role in immune cell trafficking [16,17,18]. S1PR_1_ signaling has been implicated in regulatory T-cell (Treg) development and function, with receptor modulation promoting immune tolerance, a mechanism relevant to autoimmune conditions such as multiple sclerosis (MS) [18,20]. Given the role of the S1P–S1PR axis in immune regulation and CNS function, pharmacologic modulation of this signaling axis emerged as a therapeutic strategy in immune-mediated diseases.

**Figure 1:**
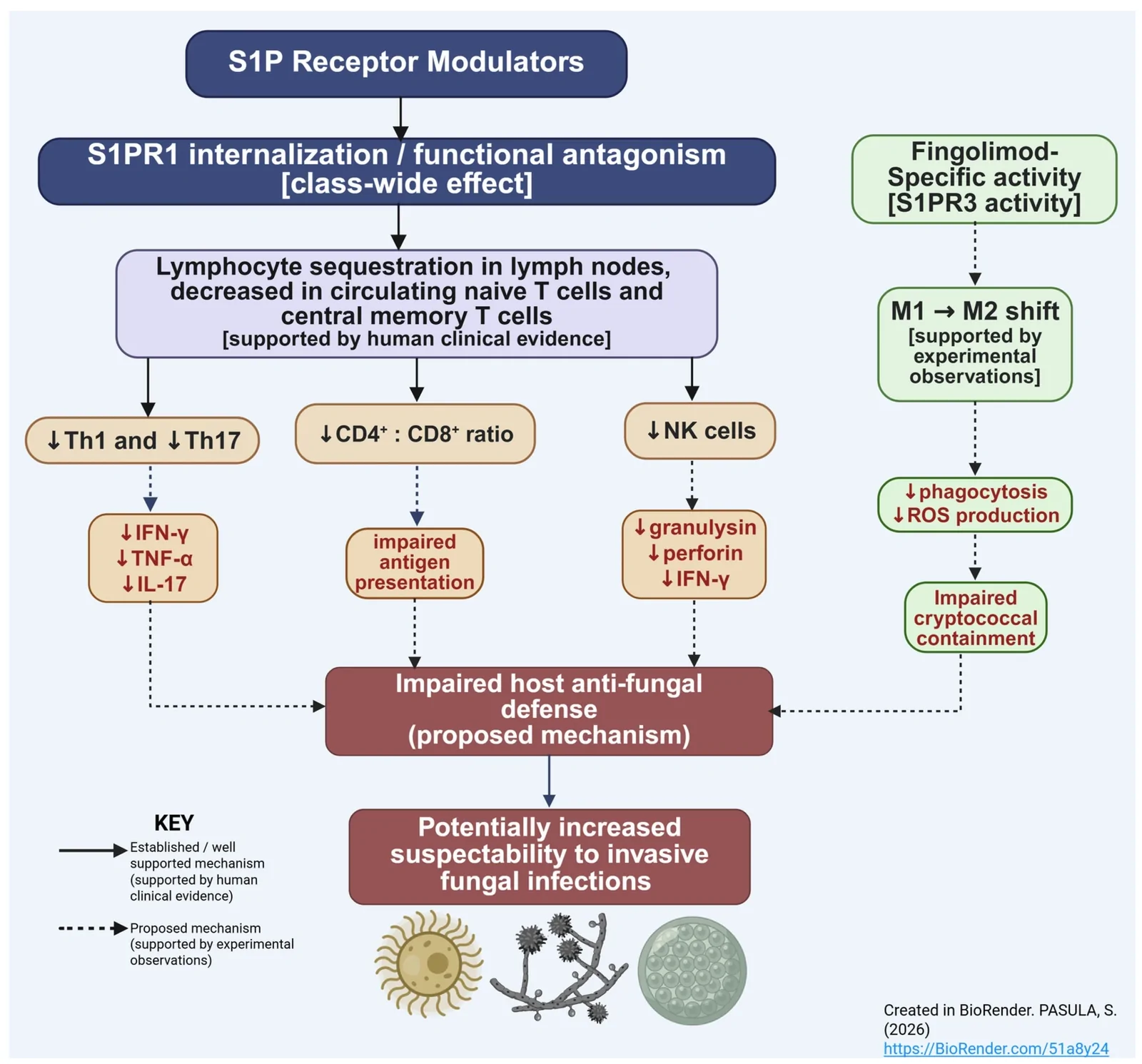
Mechanism of immunomodulation by S1P receptor modulators and susceptibility to invasive fungal infections. Abbreviations: S1P, Sphingosine 1-phosphate; S1PR, Sphingosine 1-phosphate receptor; Th1/Th17, T cell helper 1/T helper 17 cells; NK cells, Natural killer cells; CD4+, cluster of differentiation 4 positive T cell; CD8+, cluster of differentiation 8 positive T cell; ROS, Reactive oxygen species; IFN- γ, Interferon- γ; TNF-α, Tumor necrosis factor- α; IL, Interleukin; ↓, reduced. Created in BioRender. PASULA, S. (2026) https://BioRender.com/51a8y24 (accessed on 13 August 2026).

Fingolimod (FTY720; GilenyaTM, Novartis Pharma AG, Basel, Switzerland) is the first oral S1P receptor modulator to be approved for the treatment of MS [18,19,20,21]. Fingolimod is a structural analog of sphingosine and a functional antagonist of multiple S1P receptors, including S1PR_1_, S1PR_3_, S1PR_4,_ and S1PR_5_ [19,20]. Its principal immunologic effect is mediated through S1PR_1_, where sustained receptor engagement induces receptor internalization and functional antagonism, leading to CC-chemokine receptor 7 (CCR7)-mediated retention of CCR7+ T cells in lymph nodes [17,18,19,22]. This receptor modulation consequently reduces circulating CD4+ and CD8+ T-cell counts by approximately 60–80%, with a preferential reduction in naive and central memory T (T_CM_) cells due to sequestration within secondary lymphoid organs (SLO) [22]. Relative preservation or proportional increase in effector memory T (T_EM_) cells is observed due to the lack of CCR7 expression and predominant residence in non-lymphoid peripheral tissues rather than SLO [22]. This redistribution leads to peripheral lymphopenia, reducing the recirculation of potentially autoreactive lymphocytes and preventing their infiltration into the central nervous system, without direct T-cell depletion, typically evident within the first week of therapy (Figure 1) [22].

The discovery of fingolimod marked an important transition from nonspecific immunosuppression to controlled regulation of lymphocyte trafficking through S1P signaling [18]. However, fingolimod’s interaction with S1PR_3_ can cause cardiovascular effects such as transient bradycardia and self-limited atrioventricular conduction delay, which led to the development of newer, more receptor-specific drugs [23]. The newer selective S1P receptor modulators are summarized in Table 1. S1PR_1_-mediated restriction of lymphocyte egress represents the principal immunological effect shared across the drug class, whereas activity at S1PR_3_, S1PR_4,_ and S1PR_5_ varies among individual agents. In the contemporary MS treatment landscape, S1P receptor modulators are considered moderate- to high-efficacy disease-modifying therapies (DMTs), with fingolimod demonstrating approximately 50% relative reduction in relapse risk compared with placebo [16,17,24,25]. However, the infection risk associated with this broader use remains incompletely studied, and whether differences in receptor selectivity translate into clinically meaningful differences in susceptibility to fungal infections remains uncertain.

### Immunological Mechanisms Related to S1P Receptor Modulators Causing Susceptibility to Fungal Infections

There is emerging clinical evidence suggesting an association between the immunological alterations caused by S1P receptor modulators and an increased susceptibility to fungal infections [28,29,30,31,32]. Host antifungal defense depends on the coordinated activity of innate and adaptive immune responses, particularly Th1- and Th17-mediated immunity [2]. A decline in circulating T helper 1 (Th1) and T helper 17 (Th17) subsets with S1P receptor modulators may impair responses to new infections, while a reduction in central memory T cells may compromise immune surveillance [33]. Upon recognition of fungal pathogens, antigen-presenting cells, including dendritic cells and macrophages, produce IL-12 and IL-23 [34]. IL-12 promotes Th1 differentiation and interferon-gamma (IFN-γ) production, whereas IL-23 supports Th17 responses [8]. Suppression of Th1 activity reduces the production of key antifungal cytokines, including interferon-gamma (IFN-γ) and tumor necrosis factor-alpha (TNF-α), diminishing macrophage activation and fungal clearance [35]. In addition, Th17 cells are an important source of IL-17 production, which promotes neutrophil recruitment, leukocyte activation, and sustained antifungal immunity; impairment of this pathway may further compromise host defense [35]. Although these proposed immunological mechanisms provide a biologically plausible explanation for increased susceptibility to fungal infections during S1P receptor modulator therapy, much of the mechanistic evidence is derived from experimental and preclinical studies, and direct human evidence remains limited.

Among the fungal pathogens associated with impaired cell-mediated immunity, *Cryptococcus* species are of clinical relevance. *Cryptococcus neoformans* species complex and *Cryptococcus gattii* species complex are medically important species complexes, with *C. neoformans* species complex being more prevalent worldwide [1]. These encapsulated yeasts are ubiquitously present in the environment and are acquired by inhalation of propagules, leading to pulmonary infection [1,2]. This pulmonary infection can be asymptomatic, cleared, or remain latent; however, in some individuals with risk factors, dissemination may occur, most often leading to cryptococcal meningitis (CM) [1].

**Figure 2:**
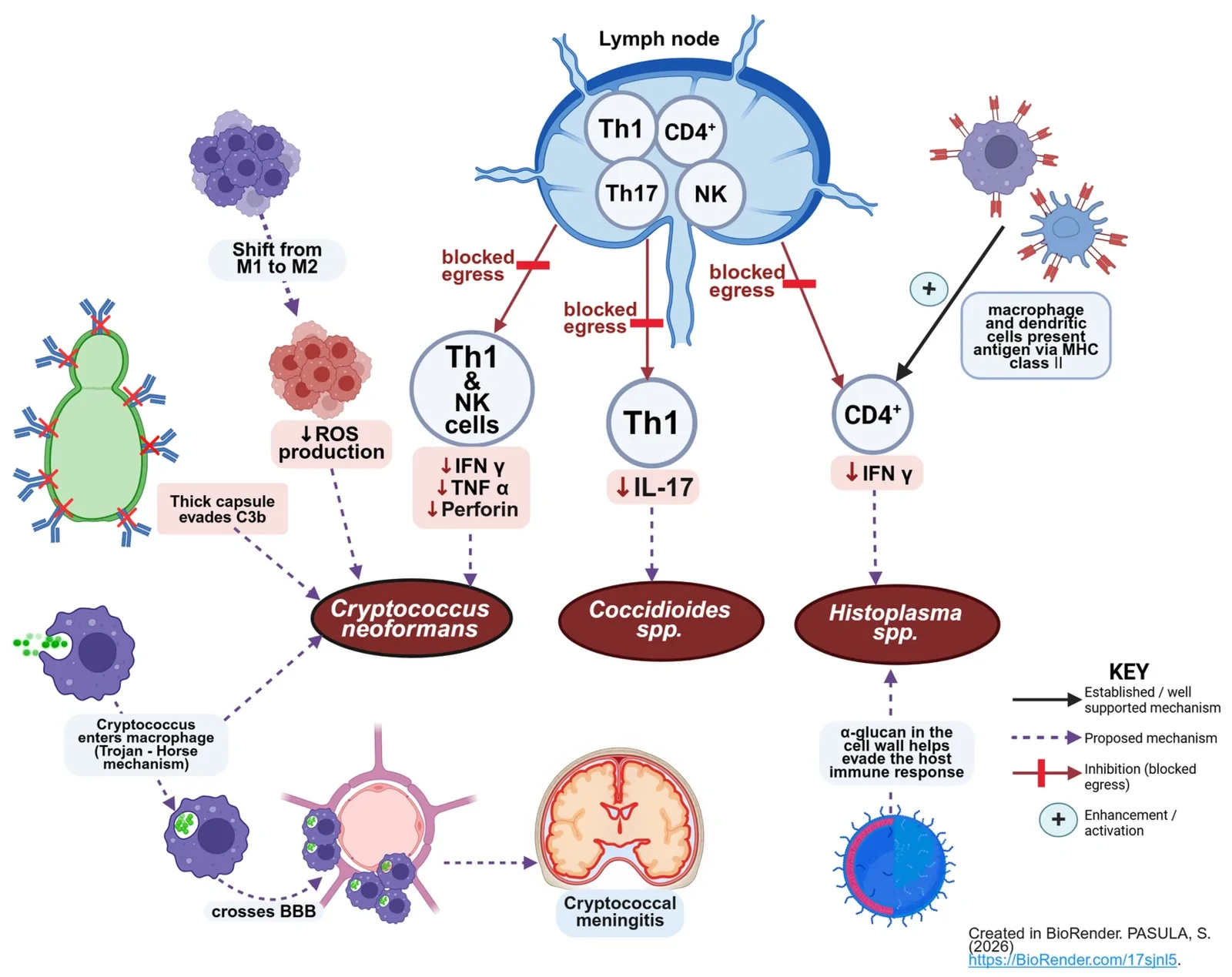
Pathogen-specific host immune interactions with *Cryptococcus, Histoplasma* and *Coccidioides* during S1P receptor modulator use. Abbreviations: Th1/Th17, T cell helper 1/T helper 17 cells; NK cells, Natural killer cells; CD4+, cluster of differentiation 4 positive T cell; ROS, Reactive oxygen species; IFN- γ, Interferon- γ; TNF-α, Tumor necrosis factor- α; IL, Interleukin; MHC class II, Major histocompatibility complex class I; C3b, Complement component 3b; BBB, blood–brain barrier; spp, species; ↓, reduced. Created in BioRender. PASULA, S. (2026) https://BioRender.com/17sjnl5 (accessed on 13 August 2026).

*Cryptococcus* employs multiple mechanisms to evade host immune defenses and exploit defects in cellular immunity. The thick polysaccharide capsule is the key virulence factor [36,37]. The capsule can activate the alternative complement pathway; however, it limits effective immune clearance by reducing C3b deposition on its surface, thereby impairing opsonization and subsequent phagocytosis by immune effector cells, namely polymorphonuclear leucocytes and monocytes [36,38,39] (Figure 2). Furthermore, during infection, *Cryptococcus* increases its capsule size, which moves the complement away from the cell surface and further reduces opsonization [37]. Therefore, capsule size and composition are key determinants of opsonization [37].

In addition to its extracellular defenses, *Cryptococcus* can survive within macrophages by altering phagosomal pH and resisting intracellular killing [37]. Rather than being destroyed, it uses macrophages as a protective intracellular niche for replication [35]. This process, known as the “Trojan horse” mechanism, allows infected macrophages to traffic the organism across the blood–brain barrier (BBB), facilitating central nervous system dissemination [35,37] (Figure 2).

Moreover, *Cryptococcus* modulates immunity, shifting the response from a protective Th1 response to a Th2-dominant response [35]. Th2 polarization may suppress Th1-associated IFN- γ production and promote M2-like macrophage activation, thereby impairing cryptococcal clearance. This immunologic shift becomes significant in the setting of immunomodulatory therapies. S1PR_3_ signaling also influences macrophage polarization from M1 to M2 [40]. Macrophages are classified into two phenotypes, M1 and M2 types [41]. M1 macrophages are pro-inflammatory and eliminate fungal pathogens, while M2 macrophages support tissue repair and have reduced immune responses [41]. A balance between M1 and M2 phenotypes is important for effective control of fungal infection [41]. Thus, in the context of immunomodulation, fingolimod, through S1PR_3_ signaling was found to shift the mechanism from protective M1 macrophages to non-protective M2 macrophages, which may lead to decreased immune response to fungal infection [40] (Figure 2).

Turning to natural killer (NK) cells, they are also known to affect host defense against *Cryptococcus* directly and indirectly [35,42] (Figure 2). NK cells secrete granulysin and perforin to directly lyse the fungal pathogens [35]. Indirectly, NK cells act by secreting TNF-α and IFN-γ to enhance Th_1_ responses and boost macrophage-mediated anti-cryptococcal activity through IFN-γ and nitric oxide (NO) synthesis [35,42]. S1PR_5_ signaling contributes to NK-cell trafficking and function. Modulation of S1PR_5_ by agents with activity at this receptor may impair NK-cell-mediated antifungal immunity and plausibly contribute to susceptibility to cryptococcal infections [35,43].

T regulatory (Treg) cells are another subset of CD4+ cells targeted by S1P receptor modulators, which help suppress autoimmunity. Treg cells upon activation can suppress Th2 response and may help control cryptococcal infection, although the precise mechanism is incompletely understood [18,23].

Ceramide is a bioactive sphingolipid that participates in immune regulation through effects on inflammatory signaling, cytokine production, immune cell activation, cellular stress responses, apoptosis, and membrane organization [44]. Ceramide also serves as a metabolic precursor of sphingosine and sphingosine-1-phosphate; therefore, its immunologic effects should be considered within the broader interconnected sphingolipid metabolic network [44]. The Berdyshev et al. 2009 study is an in vitro human endothelial cell experiment showing that FTY720 inhibits multiple ceramide synthases and increases dihydrosphingosine-1-phosphate, regulating ceramide levels [45]. The Lahiri et al. 2009 paper is also an in vitro biochemical study demonstrating that FTY720 inhibits ceramide synthesis through mixed uncompetitive and noncompetitive mechanisms [46]. Together, these findings show that ceramide enhances immune activation, while fingolimod suppresses it by blocking ceramide production and shifting sphingolipid signaling toward more immunosuppressive metabolites.

Differences in receptor selectivity among S1P receptor modulators may contribute to distinct effects on antifungal immunity. Fingolimod acts on S1PR_1_, S1PR_3_, S1PR_4,_ and S1PR_5_, whereas siponimod is more selective for S1PR_1_ and S1PR_5_ [47]. S1PR_3_ signaling in macrophages is essential for proper functioning, particularly for phagocytosis [43]. In a mouse model of cryptococcal granuloma reactivation, both fingolimod and siponimod reduced circulating and pulmonary CD4+ and CD8+ T-cell populations; however, only fingolimod was associated with reduced survival, increased pulmonary and cerebral fungal burden, disruption of granuloma organization, and M2- like macrophage polarization [40]. Specifically, fingolimod reactivated cryptococcosis from granulomas through a proposed S1PR_3_ mediated mechanism involving impaired macrophage phagocytosis and reduced reactive oxygen species (ROS) production [40]. This mechanistic difference suggests that fingolimod-mediated disruption of S1PR3 signaling may impair macrophage control of latent cryptococcal infection. However, these observations are derived from experimental models and do not establish a difference in clinical risk between fingolimod and siponimod. The greater number of published cryptococcal infections associated with fingolimod may also be due to its longer duration of clinical use, greater cumulative exposure, and/or reporting differences, and does not establish a higher risk compared with newer S1P modulators.

*Histoplasma capsulatum* has its own ways to evade the immune system; one such way is through cell wall components [48]. After infecting the host, *Histoplasma* increases alpha-glucan production, produces Eng1, which hydrolyses beta-glucan, and produces melanin in its cell wall; together, they evade the host immune response [48,49,50,51]. Furthermore, *Histoplasma* infection and antigen presentation by dendritic cells and macrophages via major histocompatibility complex (MHC) class II, stimulate interleukin-12 (IL-12) production, which promotes the differentiation of naïve T cells into Th1 cells [48]. These Th1 cells subsequently secrete IFN-γ and TNF-α, enhancing macrophage activation and facilitating intracellular fungal clearance [48]. A reduction in CD4+ T cells, especially naïve T cells and CD8+ T cells, with S1P receptor modulators may therefore impair this pathway, increasing susceptibility to histoplasmosis [30,48,52] (Figure 2).

Similarly, these immunological changes impact host defense against other fungal pathogens. For instance, defense against *Coccidioides* is predominantly mediated by CD4+ T cells, particularly Th1- and Th17-driven cytokine responses, including IFN-γ, TNF-α, and IL-17 [52,53,54]. These cytokine responses facilitate macrophage activation and neutrophil recruitment, both of which are essential for effective fungal clearance [53]. Disruption of these T-cell–mediated pathways may increase the risk of severe or disseminated coccidioidomycosis (Figure 2).

## 3. Overview of S1P Receptor Modulator-Associated Infections

S1P receptor modulators are associated with a broad spectrum of infectious complications, including viral, fungal, bacterial, and protozoal infections, reflecting their immunomodulatory effects on lymphocyte trafficking. Among these, viral infections are most frequently reported, followed by fungal infections and, rarely, bacterial and protozoal infections. The most common viral infections were herpes simplex and varicella-zoster (VZV) reactivation [55,56]. In early-phase 2 and 3 clinical trials of fingolimod, the incidence of herpes virus infections was comparable between fingolimod and placebo or other DMTs [55]. In contrast, phase 3 trials of siponimod use were associated with a higher frequency of herpesvirus infections compared to placebo [55]. Phase 3 trials of ozanimod and ponesimod also report herpes virus infections [55]. Beyond herpesviruses, fingolimod is also associated with other viral infections, including activation of the JC virus (causing progressive multifocal leukoencephalopathy, PML), SARS-CoV-2, human papilloma virus (presenting as warts), HHV-8 (associated with Kaposi sarcoma), cytomegalovirus (CMV) encephalitis, hepatitis E, flaviviruses (which cause dengue fever), and poxviruses (which cause molluscum contagiosum and monkeypox) [57,58,59,60,61,62,63,64,65,66,67,68,69].

Bacterial infections with fingolimod include *Listeria* encephalitis, tubercular meningitis, and disseminated tuberculosis [70,71,72]. Protozoal infections reported to date in association with fingolimod include cerebral toxoplasmosis and visceral leishmaniasis [73,74].

Numerous fungal infections have been described in association with S1P receptor modulators, including cryptococcosis, histoplasmosis, and coccidioidomycosis. A comprehensive summary of these reported infections is presented below.

## 4. Summary of Reported Fungal Infections

We searched the PubMed database from 8 February 2026 through 15 May 2026 using the key terms (sphingosine 1 receptor modulator OR S1P receptor modulator OR fingolimod OR siponimod OR ozanimod OR ponesimod OR etrasimod) AND (fung* infection OR cryptococc* OR histoplasm* OR coccidio* OR aspergill* OR Pneumocystis), which resulted in 43 documented case reports/abstracts and one case series of 60 patients demonstrating fungal infections in patients on S1P receptor modulators, including cryptococcosis, histoplasmosis, and coccidioidomycosis. No additional databases were searched. The included reports were published between June 2014 and June 2025. Inclusion criteria were non-*Candida* fungal infections in patients receiving S1P receptor modulators. Non-English-language papers and reports of candidal infections were excluded. Fingolimod accounted for most of the identified cases, whereas only 2 cases were reported in patients on siponimod. Our literature search identified no published cases of invasive fungal infections in patients receiving ozanimod, ponesimod, or etrasimod, and no reported cases of invasive aspergillosis or *Pneumocystis jirovecii* pneumonia associated with S1P receptor modulator therapy. As this is a narrative review based primarily on a PubMed search; it was not intended to be a systemic review and may therefore be subject to publishing, indexing, and reporting biases. We identified one duplicate case report, which was subsequently excluded. Regarding the case series, the 60 patients were obtained from the Novartis safety database, and we cannot exclude the possibility of any duplicates with the other 43 individual case reports/abstracts. Incomplete data in abstracts and case reports were documented as “not reported” in the tables.

Among patients receiving fingolimod, the literature search identified 37 individual case reports and one 60-patient case series of cryptococcal infection, as well as three histoplasmosis and one coccidioidomycosis. Of the cryptococcal infections, eight cases were found to have disseminated disease. Nineteen individual cases along with the 60-patient case series involved cryptococcal meningitis/meningoencephalitis. Six cases were classified as primary cutaneous cryptococcosis/cutaneous cryptococcoma; two cases as pulmonary cryptococcosis (one of which was asymptomatic); and two cases as cryptococcal osteomyelitis.

Among the eight cases of disseminated cryptococcosis with or without CNS involvement, age was reported in 8/8 patients and ranged from 20 to 63 years; 75% (6/8) were male (Table S1). Fingolimod treatment duration was reported in 8/8 patients and ranged from 16 months to 8 years. Six out of eight patients reportedly had prior exposure to other DMTs/immunosuppressive agents, including one patient who had previously received chemotherapy. Six out of eight were tested for HIV, and all of them were negative; additionally, one of these five was IgG2 deficient. The absolute lymphocyte count (ALC) at presentation was reported in 6/8 patients, with available values ranging from 0.09 × 10^9^/L to 0.5 × 10^9^/L. The absolute CD4+ count at presentation was reported in 5/8 patients, with available values ranging from 61 to 145 cells/mm^3^. Antifungal treatment was reported for 8/8 patients and typically consisted of liposomal amphotericin B and flucytosine, followed by fluconazole, although one patient received amphotericin and fluconazole without flucytosine, and another patient received fluconazole alone. MS medication modification was reported in 6/8 patients, and fingolimod was discontinued in all six. Whether fingolimod was continued or discontinued was not reported in the remaining two patients. All patients reportedly recovered, although one patient with CNS involvement had only partial improvement in lower extremity muscle weakness and cognitive impairment [75,76,77,78,79,80,81,82].

Nineteen individual cases and one 60-patient case series described cryptococcal meningitis or meningoencephalitis (Table S2). Among the individual cases, age was reported in 19/19 patients and ranged from 21 to 67 years; 57.9% (11/19) were female. Fingolimod treatment duration was reported for 18/19 patients and ranged from 2 to 12 years. Prior exposure to other DMTs/immunosuppressive agents was documented in 9/19 patients. Of these, 5/9 had previously received IFN Beta, 3/9 had previously received natalizumab, and 4/9 had previously received multiple medications. HIV testing was reported for 9/19 patients, all of whom were negative. The absolute lymphocyte count at presentation was reported for 17/19 patients, with values ranging from 0.12 × 10^9^/L to 2.89 × 10^9^/L. The absolute CD4+ counts were reported for 7/19 patients and ranged from 5 to 667 cells/mm^3^; an additional patient was described as having a normal CD4+ count, although no numerical value was provided [83]. Antifungal treatment was reported for 18/19 patients and commonly treated with amphotericin B, with or without flucytosine, followed by fluconazole. Serial therapeutic lumbar punctures, EVDs, and VP shunts were used when clinically indicated. MS medication modification was reported in 14/19 patients, with fingolimod reportedly discontinued in 13/14 patients. Outcomes were reported in 18/19 patients: 16 recoveries and 2 deaths. Immune reconstitution inflammatory syndrome developed in 5/16 patients who recovered [35,83,84,85,86,87,88,89,90,91,92,93,94,95,96,97,98,99,100].

In the 60-patient case series of cryptococcal meningitis/meningoencephalitis, age was available for 52/60 patients and ranged from 22 to 67 years; 51.8% (29/56) were female (Table S2). Fingolimod treatment duration was reported in 44/60 patients and ranged from 1 to 9 years. Medical history was documented in 28/60 patients, 26 of whom received some previous unspecified MS therapy. HIV status and CD4 counts were not reported. Lymphocyte-count data were available for 31/60 patients: 8/31 had <0.200 × 10^9^/L, 17/31 had 0.200 to <0.500 × 10^9^/L, 4/31 had 0.500 to <0.800 × 10^9^/L, and 2/31 had values within the normal range. Treatment was reported in 36/60 patients. Of these, 31/36 received systemic antifungal therapy with amphotericin B, flucytosine, and fluconazole, whereas 5/36 received an unspecified antifungal regimen. MS medication modification was not reported. Outcomes were available in 43/60 patients: 26 recovered, 13 deaths, one deteriorating, and three remain unchanged [28]. Although the demographic characteristics and duration of fingolimod exposure appeared broadly similar between the individual reports and the case series, substantial missing data—particularly regarding CD4+ counts, antifungal treatment, and fingolimod modification—limited direct comparisons.

Among the six patients with primary cutaneous cryptococcosis and cutaneous cryptococcoma, age was reported in 6/6 patients and ranged from 33 years to 63 years; 66.7% (4/6) were females (Table S3). Fingolimod treatment duration was reported in 6/6 patients and ranged between 2 and 9 years. Only 1/6 patients reportedly received other DMTs/immunosuppressive therapies. HIV testing was reported for 5/6 patients; all of them were negative. The absolute lymphocyte count at presentation was reported in 5/6 patients and ranged from 0.30 × 10^9^/L to 1.3 × 10^9^/L. The absolute CD4+ count at presentation was reported in 3/6 patients and ranged from 13 cells/mm^3^ to 91.7 cells/mm^3^. Antifungal treatment and MS medication modification was reported in 5/6 cases and typically consisted of discontinuing fingolimod and starting oral fluconazole; however, the patient with cutaneous cryptococcoma was treated with tumor excision along with amphotericin and flucytosine, followed by oral fluconazole with flucytosine [101]. Fingolimod was subsequently resumed in one patient. Outcomes were reported in 5/6 patients, all of whom recovered completely [101,102,103,104,105,106].

Of the two pulmonary cryptococcosis cases (Table S3), detailed information was only available for one patient. This patient was a 45-year-old male who had received fingolimod for three years and had prior exposure to corticosteroids and IFN beta-1a/1b. His ALC at presentation was 0.680 × 10^9^/L. The patient was treated with fluconazole, resulting in partial recovery. Fingolimod was discontinued, and treatment for multiple sclerosis was subsequently changed to glatiramer acetate (and later dimethyl fumarate) [107,108].

Two cases involving cryptococcal osteomyelitis were identified: necrotizing cryptococcal osteomyelitis and a cryptococcal chest-wall mass with rib osteomyelitis (Table S3). Age was reported for both patients and ranged from 43 to 46 years; both were female. Fingolimod treatment duration was reported for both cases and ranged from 4 to 11 years and eight months. The absolute lymphocyte count at presentation was reported for both patients and ranged from 0.3 × 10^9^/L to 1.2 × 10^9^/L. Treatment was reported for both patients. The patient with necrotizing cryptococcal osteomyelitis received voriconazole, whereas the patient with a cryptococcal chest-wall mass and rib osteomyelitis received liposomal amphotericin B and flucytosine followed by fluconazole. Both patients achieved complete recovery. MS medication modification was reported in one patient, in whom fingolimod was discontinued [29,109].

Among the three patients with histoplasmosis while on fingolimod, the ages ranged from 30 to 54 years; 66.7% (2/3) were females (Table 2). Fingolimod treatment duration was reported in 2/3 patients and ranged from 1 to 3 years. The absolute lymphocyte count at presentation was reported for one patient and it was 1.17 × 10^9^/L. Antifungal treatment was reported for all of the patients. Of the two patients with disseminated histoplasmosis, one received amphotericin B followed by itraconazole, although the clinical outcome was not reported, and the other received itraconazole and achieved complete recovery. The patient with primary cutaneous histoplasmosis also received itraconazole and achieved complete recovery. MS medication modification was reported in 2/3 patients, and fingolimod was stopped in both. One patient with disseminated histoplasmosis was subsequently switched to natalizumab [30,32,110].

The single reported case of disseminated coccidioidomycosis involved a 56-year-old female on fingolimod for 16 years for the treatment of multiple sclerosis (Table 2). She had meningitis, cellulitis, and osteomyelitis. She was treated with amphotericin B and voriconazole, underwent irrigation and debridement of ankles, and was transitioned to oral anti-fungal medication, resulting in partial recovery [31].

There have been two documented cases of cryptococcal meningitis in patients treated with siponimod (Table 3). Of these patients, information was available for only one, a 61-year-old male treated with amphotericin B followed by fluconazole, leading to complete recovery [111]. There has been one documented case that occurred during a phase III clinical trial, information on which was not provided within the body of the manuscript nor in the Supplemental Materials [112].

## 5. Risk Mitigation and Clinical Implications:

Compared to the overall population of MS patients receiving S1P receptor modulators, the incidence of IFIs remains low; however, current evidence is mostly limited to case reports, case series, and small observational cohorts, which may underestimate the true incidence. Most of the cases published to date have been associated with fingolimod use; very few cases have been associated with siponimod use. The absence of published cases involving newer S1P receptor modulators may reflect their recent introduction into clinical practice, shorter cumulative exposure and/or underreporting. Establishing a global surveillance network may help better define the true burden and spectrum of IFIs associated with S1P receptor modulators. In the meantime, clinicians can consider employing individualized risk assessment, including screening and monitoring strategies pre-treatment and during treatment with S1P receptor modulators.

Pretreatment evaluation before initiating S1P receptor modulator therapy should include a comprehensive clinical assessment and establishment of relevant laboratory baselines [9,113,114]. The clinical assessment should include evaluation for active infection, and S1P receptor modulator therapy should not be initiated in patients with an active infection [114]. Assessment of epidemiologic exposures relevant to endemic fungal infections, history of organ transplantation, and other comorbidities such as HIV may be considered based on the patient’s history and risk factors. Prior and concomitant disease-modifying or immunosuppressive therapies should also be reviewed because of the potential for additive immunosuppressive effects. A recent complete blood count (CBC) with differential and liver function tests (LFTs) should be obtained before treatment initiation to identify pre-existing lymphopenia or hepatic abnormalities and to provide a baseline for subsequent monitoring [9,113]. Varicella-zoster virus immunity should be documented; vaccination of sero-negative patients is recommended before initiating S1P receptor modulators [114,115,116]. Additional assessments can include a baseline skin examination and an ophthalmological evaluation [9].

During treatment with an S1P receptor modulator, patients should be monitored clinically for infection, with particular attention to any new neurologic, pulmonary, or dermatologic symptoms [114,115,116]. Any new neurologic symptoms in this population warrant careful evaluation as cryptococcal infections can closely mimic MS exacerbations; failure to distinguish between the two may delay antifungal therapy and prompt unnecessary immunosuppression. Thus, a multidisciplinary approach may be appropriate in suspected cases [107]. Periodic assessment of the CBC with differential may be considered to monitor lymphopenia, which may contribute to susceptibility to infection. Patients older than 45 years and those on therapy for more than two years were observed to have an association with IFIs; however, these observations are derived from case reports and should not be interpreted as established risk factors [107].

When an invasive fungal infection is suspected, prompt diagnostic evaluation should be guided by the presenting syndrome, potential organ involvement, and relevant epidemiological exposures. Depending on the clinical presentation, evaluation may include CBC, CMP, serum cryptococcal antigen, blood and/or sputum cultures, chest computed tomography, magnetic resonance image of the brain, and/or tissue biopsy. When cryptococcal meningitis is suspected, lumbar puncture should include CSF opening pressure, protein, glucose, cell counts, microscopy, cultures, and CSF cryptococcal antigen testing. Serum or urine *Histoplasma* antigen and *Coccidioides* serologies may be added depending on the clinical presentation and epidemiological risk factors. Antifungal treatment should be guided by the clinical syndrome and disease severity in accordance with the European Confederation of Medical Mycology (ECMM)/International Society for Human and Animal Mycology (ISHAM) global guidelines developed in cooperation with ASM and endorsed by the Infectious Diseases Society of America (IDSA) [117]. Patients with disseminated cryptococcosis and cryptococcal meningitis/meningoencephalitis generally require induction therapy with liposomal amphotericin B and flucytosine, followed by fluconazole for consolidation and maintenance therapy [117]. Isolated pulmonary cryptococcosis and primary cutaneous cryptococcosis should be managed depending upon disease severity; severe disease requires systemic therapy, and mild-to-moderate disease requires oral antifungal therapy [117]. Likewise, treatment of histoplasmosis and coccidioidomycosis should be individualized to disease severity, extent of dissemination, host immune status, and current guideline recommendations.

The decision regarding modification of MS therapy should be individualized. Although fingolimod was discontinued or switched to an alternative DMT in many published cases with available treatment information, these reports do not establish a standardized approach. The potential benefits of treatment interruption should be weighed against the risks of immune reconstitution inflammatory syndrome, MS flare, and severe disease rebound following treatment withdrawal. Multidisciplinary decision-making should be employed after thorough deliberation between neurology and infectious disease specialists.

There are currently no established guidelines for antimicrobial prophylaxis in immunosuppressed patients on S1P receptor modulators. The most recent joint guidelines from ECMM and ISHAM, endorsed by IDSA, do not recommend primary prophylaxis for cryptococcal infections in HIV+ patients without a positive serum cryptococcal antigen test [117]. Similarly, in non-HIV immunocompromised patients, routine serum cryptococcal antigen testing, prophylaxis, and pre-emptive therapy are not currently recommended [117]. Current evidence is limited to the general immunocompromised population; therefore, future multicenter prospective studies are required to evaluate these strategies specifically in patients receiving S1P receptor modulators. Additionally, further research is needed to compare the risk among various S1P receptor modulators; identify patient-specific risk factors, including absolute lymphocyte count and absolute CD4+ count; translational immunological research for better understanding of the host–pathogen interaction in the setting of S1P receptor modulators; and screening, antifungal prophylaxis, and safe resumption of medication following infection.

## 6. Conclusions

Invasive fungal infections continue to represent serious infections worldwide, especially with the growing number of immunocompromised patients, including those on immunomodulatory therapies, such as the S1P receptor modulators for MS. Among the various medications in this class, fingolimod has the largest number of documented cases of IFIs. Most of these infections represent cryptococcal infections, in addition to histoplasmosis and coccidioidomycosis. Given the potential severity of these infections, clinicians should consider baseline patient-specific risk factors and remain attentive to clinical features suggestive of fungal infection during therapy. A high clinical suspicion must be maintained when MS patients on S1P receptor modulators present with new or worsening respiratory, neurological, or dermatological complaints, as early recognition, diagnostic evaluation, and appropriate treatment are crucial for reducing morbidity and mortality.

## Figures and Tables

**Table 1:** FDA-approved S1P Receptor Modulators for the Treatment of MS and their Receptor Selectivity.

Drug	Receptor Selectivity	Year of FDA Approval for MS
Fingolimod [9,20]	S1PR_1_, S1PR_3_, S1PR_4,_ and S1PR_5_	2010
Siponimod [9,26]	S1PR_1_ and S1PR_5_	2019
Ozanimod [9,27]	S1PR_1_ and S1PR_5_	2020
Ponesimod [9]	S1PR_1_	2021

Abbreviations: S1P, Sphingosine 1-phosphate; S1PR, Sphingosine 1-phosphate receptor; MS, multiple sclerosis; FDA, U.S. Food and Drug Administration.

**Table 2:** Reported Cases of Histoplasmosis and Coccidioidomycosis in Patients Receiving Fingolimod.

Study	Age/ Sex	Fingolimod Duration in Years (Months)	Prior DMT/ Immunosuppression	HIV Status	Diagnosis	ALC at Presentation (Cells × 10^9^/L)	Absolute CD4+ Count at Presentation (Cells/mm^3^)	Antifungal Treatment	MS Medication Modification	Outcome
Abrahamowicz et al. (2021) [30]	46/M	NR	None	Negative	Disseminated Histoplasmosis (Liver and GI)	NR	NR	L-AmB → Itraconazole	NR	NR
de Oliveira et al. (2024) [110]	30/F	1 (12)	NR	NR	Disseminated Histoplasmosis (Lung, Spleen, Lymph Nodes)	1.17	NR	Itraconazole	Switched to Natalizumab	Complete recovery
Veillet-Lemay et al. (2017) [32]	54/F	3 (36)	NR	NR	Primary Cutaneous Histoplasmosis	NR	NR	Itraconazole	Discontinued	Complete recovery
Denton et al. (2024) [31]	56/F	16 (192)	NR	NR	Disseminated Coccidioidomycosis (CNS, Skin, and Bone)	NR	NR	AmB and Voriconazole → Itraconazole → Fluconazole → Posaconazole I&D of both ankles	Resumed *	Partial recovery

Abbreviations: L-AmB, Liposomal Amphotericin B; AmB, Amphotericin B; DMT, disease modifying therapy; ALC, absolute lymphocyte count; HIV, human immunodeficiency virus; MS, multiple sclerosis; CNS, central nervous system; I&D, irrigation and debridement; GI, gastrointestinal; NR, not reported. * Stopped 8 months before presentation.

**Table 3:** Reported Cases of Invasive Fungal Infections in Patients Receiving Siponimod.

Study	Age/ Sex	Siponimod Duration in Years (Months)	Prior DMT/ Immunosuppression	HIV Status	Diagnosis	ALC at Presentation (Cells × 10^9^/L)	Absolute CD4+ Count at Presentation (Cells/mm^3^)	Antifungal Treatment	MS Medication Modification	Outcome
Manser et al. (2024) [111]	61/M	NR	NR	NR	Cryptococcal Meningitis	NR	NR	AmB → Fluconazole	Discontinued	Complete Recovery
Cree et al. (2022) [112]	NR	NR	NR	NR	Cryptococcal Meningitis	NR	NR	NR	NR	NR

Abbreviations: AmB, Amphotericin B; DMT, disease modifying therapy; ALC, absolute lymphocyte count; HIV, human immunodeficiency virus; MS, multiple sclerosis; NR, not reported.

## Data Availability

No new data were created in this study. The information summarized in this review was obtained from the published literature cited in the article, and the extracted information is presented within the article and its tables.

## Supplementary Materials

The following supporting information can be downloaded at https://www.mdpi.com/article/10.3390/microorganisms14081861/s1: Table S1: Reported Cases of Disseminated Cryptococcal Infections with and without CNS Involvement in Patients Receiving Fingolimod. Table S2: Reported Cases of Cryptococcal Meningitis/Meningoencephalitis in Patients Receiving Fingolimod. Table S3: Reported Cases of Other Cryptococcal Infections in Patients Receiving Fingolimod.

## Abbreviations

The following abbreviations are used in this manuscript:

ALC	Absolute lymphocyte count
AmB	Amphotericin B
BBB	Blood–Brain Barrier
CBC	Complete Blood Count
CCR7	CC-Chemokine receptor 7
CM	Cryptococcal Meningitis
CMV	Cytomegalovirus
CSF	Cerebrospinal Fluid
DMT	Disease-Modifying Therapy
ECMM	European Confederation of Medical Mycology
EVD	External Ventricular Drain
FDA	Food and Drug Administration
FTY720	Fingolimod
GI	Gastrointestinal
HIV	Human Immunodeficiency Virus
HHV-8	Human Herpesvirus 8
HPV	Human Papillomavirus
IDSA	Infectious Diseases Society of America
IFI	Invasive Fungal Infection
IFN	Interferon
IFN-γ	Interferon Gamma
IgG2	Immunoglobulin G Subclass 2
IL	Interleukin
IRIS	Immune Reconstitution Inflammatory Syndrome
L-AmB	Liposomal Amphotericin B
JC Virus	John Cunningham Virus
LFT	Liver Function Test
MHC	Major Histocompatibility Complex
MS	Multiple Sclerosis
NK	Natural Killer
NR	Not Reported
PML	Progressive Multifocal Leukoencephalopathy
ROS	Reactive Oxygen Species
SARS-CoV-2	Severe Acute Respiratory Syndrome Coronavirus 2
S1P	Sphingosine-1-Phosphate
S1PR	Sphingosine-1-Phosphate Receptor
S1PR1	Sphingosine-1-Phosphate Receptor 1
S1PR3	Sphingosine-1-Phosphate Receptor 3
S1PR4	Sphingosine-1-Phosphate Receptor 4
S1PR5	Sphingosine-1-Phosphate Receptor 5
SLO	Secondary Lymphoid Organs
TEM	Effector Memory T Cell
Th1	T Helper 1 Cell
Th2	T Helper 2 Cell
Th17	T Helper 17 Cell
TNF-α	Tumor Necrosis Factor Alpha
Treg	Regulatory T Cell
VP Shunt	Ventriculoperitoneal Shunt
VZV	Varicella-Zoster Virus
