# Supplementary material for: Fungal Infections Associated with Sphingosine 1-Phosphate Receptor Modulators: Immunological Mechanisms, Clinical Patterns, and Management Considerations
Source: Microorganisms. 2026 Aug 21;14(8):1861. doi: 10.3390/microorganisms14081861 (PMC13515163; doi:10.3390/microorganisms14081861)
Supplement: Supplementary file 1 [file microorganisms-14-01861-s001.zip › microorganisms-4440988-supplementary.pdf]

[Table S1] Reported Cases of Disseminated Cryptococcal Infections with and without CNS Involvement in Patients Receiving Fingolimod

Study	Age/Sex	Fingolimod Duration in Years (Months)	Prior DMT/Immunosuppression	HIV Status	Organ Involvement	ALC at Presentation (cells × 10^9/L)	Absolute CD4+ Count at Presentation (cells/mm^3)	Antifungal Treatment	MS Medication Modification	Outcome
**Disseminated Cryptococcosis with CNS Involvement**										
Kaur et al. (2020) [75]	34/M	5 (60)	*NR*	Negative	CNS and Fungemia	*NR*	61	Fluconazole → L-AmB and Flucytosine → Fluconazole	*NR*	Complete recovery
Seto et al. (2016) [76]	63/M	2 (24)	Unspecified DMTs	Negative	CNS, Skin, and Lung	0.3	145	L-AmB and Flucytosine → Fluconazole	Discontinued	Complete/partial recovery*
Huang (2015) [77]	50/M	3.5 (42)	IFN Beta-1a	Negative	CNS and Skin	0.50	*NR*	AmB and Flucytosine → Fluconazole	*NR*	Complete recovery
Chey et al. (2023) [79]	56/F	3.75 (45)	Chemotherapy (Fluorouracil, Epirubicin, Cyclophosphamide and Docetaxel), followed by Tamoxifen	*NR*	CNS and Cutaneous	0.2	*NR*	AmB and Fluconazole	Discontinued<br><br>Peginterferon Beta-1a 8 months later	Complete recovery
Wienemann et al. (2020) [81]	49/F	5.5 (66)	IFNs, Natalizumab, Mitoxantrone, and Rituximab	*NR***	CNS and Lung	0.09	77	L-AmB and Fluconazole → L-AmB and Flucytosine → Fluconazole	Discontinued	Complete recovery
**Disseminated Cryptococcosis without CNS Involvement**										
Kammeyer and Lehman (2022) [78]	61/M	7.5 (90)	Methylprednisolone	Negative	Fungemia	0.3	*NR*	L-AmB and Flucytosine → Fluconazole<br><br>60 days later, Methylprednisolone → Fluconazole	Discontinued	Complete recovery
Sharma et al. (2024) [80]	20s/M	8 (96)	*NR*	Negative	Choroiditis and Osseous Cryptococcosis	*NR*	80	L-AmB → Fluconazole and Flucytosine → Fluconazole	Discontinued	Stable vision and complete recovery of back pain
Carpenter et al. (2017) [82]	47/M	1.33 (16)	IFN Beta-1b and Dimethyl Fumarate	Negative	Primary Cutaneous Cryptococcosis and Fungemia	0.3	73	Fluconazole	Discontinued and started on Glatiramer Acetate and Methylprednisolone	Complete recovery

Abbreviations: L-AmB, Liposomal Amphotericin B; AmB, Amphotericin B; DMT, disease modifying therapy; ALC, absolute lymphocyte count; HIV, human immunodeficiency virus; MS, multiple sclerosis; CNS, central nervous system; VZV, varicella-zoster virus; IFN, interferon; *NR*, Not reported.

*Complete recovery of cutaneous lesions, partial recovery of lower extremity muscle weakness and cognitive impairment

**IgG2 Deficient

[Table S2] Reported Cases of Cryptococcal Meningitis/Meningoencephalitis in Patients Receiving Fingolimod

Study	Age/Sex	Fingolimod Duration in Years (Months)	Prior DMT/Immunosuppression	HIV Status	Diagnosis	ALC at Presentation (cells $\times 10^9$/L)	Absolute CD4+ Count at Presentation (cells/mm^3)	Antifungal Treatment	MS Medication Modification	Outcome
Zhou et al.<br>(2023) [85]	67/M	6 (72)	None	*NR*	Cryptococcal Meningoencephalitis	0.2	*NR*	*NR*	*NR*	*NR*
Ma et al.<br>(2020) [86]	58/M	7 (84)	IFN Beta-1b	Negative	Cryptococcal Meningoencephalitis	0.9	*NR*	AmB and Flucytosine → Fluconazole	Discontinued<br><br>Started on Peginterferon Beta-1a	Complete recovery
Anene-Maidoh et al.<br>(2018) [84]	61/F	4.83 (58)	Glatiramer Acetate,<br>IFN Beta-1-a	Negative	Cryptococcal Meningoencephalitis	*NR*	69	AmB and Flucytosine	*NR*	Death
Achtnichts et al.<br>(2015) [87]	40s/M	2 (24)	*NR*	*NR*	Cryptococcal Meningoencephalitis	0.40	56	L-AmB and Flucytosine → Fluconazole	Discontinued<br><br>Switched to Glatiramer Acetate	IRIS followed by complete recovery
Cuascut et al.<br>(2021) [88]	48/F	7.6 (91)	Abatacept,<br>Hydroxychloroquine		Cryptococcal Meningoencephalitis	0.21	*NR*	AmB and Flucytosine → Fluconazole<br><br>VP Shunt	Fingolimod and Abatacept Discontinued, Hydroxychloroquine continued	IRIS followed by complete recovery
Nasir et al.<br>(2023) [89]	21/F	5 (60)	Natalizumab	Negative	Cryptococcal Meningoencephalitis	0.53	6	AmB and Flucytosine → Fluconazole	Discontinued	Complete recovery
Kuruoglu et al.<br>(2019) [90]	37/M	5 (60)	High-dose corticosteroids followed by IFN Beta-1a	*NR*	Cryptococcal Meningoencephalitis	0.62	192	L-AmB and Fluconazole → Fluconazole	Discontinued	Complete recovery
Baghbanian and Amiri<br>(2020) [91]	41/F	5 (60)	*NR*	Negative	Cryptococcal Meningoencephalitis	0.25	*NR*	AmB and Fluconazole<br><br>VP shunt	Started on IFN Beta*	Complete recovery
Juusola et al.<br>(2025) [92]	46/M	5.42 (65)	High-dose steroids	Negative	Cryptococcal Meningoencephalitis	0.46	37	AmB and Fluconazole → Fluconazole<br><br>Later returned and received AmB and Flucytosine → Fluconazole	Discontinued	Gradual recovery followed by IRIS then complete recovery
Chong et.al.<br>(2019) [93]	40/F	2.25 (27)	*NR*	*NR*	Cryptococcal Meningitis	0.2	*NR*	AmB and Flucytosine	Discontinued	*NR*
Pham et al.<br>(2017) [94]	61/F	3 (36)	Natalizumab	*NR*	Cryptococcal Meningitis	0.12	5	L-AmB and Flucytosine → Fluconazole<br>EVD converted to VP shunt	Discontinued	Complete recovery

Ward et al. (2016) [95]	67/F	3.33 (40)**	IFN Beta, Natalizumab, Methylprednisolone	Negative	Cryptococcal Meningitis	2.39	*NR*	AmB → Fluconazole	*NR*	IRIS followed by death
Grebenciucova et al. (2016) [35]	62/M	3 (36)	*NR*	*NR*	Cryptococcal Meningitis	0.336	*NR*	AmB and Flucytosine → Fluconazole	Discontinued	Complete recovery
Aoki et al. (2021) [83]	41/M	6 (72)	*NR*	Negative	Cryptococcal Meningitis	0.177	Normal (but number not officially reported)	L-AmB and Flucytosine → Fluconazole	Discontinued	Complete recovery
Nakagawa et al. (2025) [96]	65/F	11.92 (143)	*NR*	*NR*	Cryptococcal Meningitis	0.520	*NR*	L-AmB and Flucytosine → Fluconazole	*NR*	Complete recovery
Darazam et al. (2022) [97]	39/F	5 (60)	IFN Beta-1a	*NR*	Cryptococcal Meningitis	0.5	*NR*	L-AmB and Fluconazole → Fluconazole<br><br>Lumbar drains	Discontinued	Complete recovery
Singh and Al-Awwad (2024) [98]	44/M	8 (96)	*NR*	*NR*	Cryptococcal Meningitis/Cerebellitis	0.250	*NR*	L-AmB and Flucytosine	*NR*	Death
Mittal and Ramos (2023) [99]	25/F	*NR*	*NR*	Negative	Cryptococcal Meningitis	*NR*	*NR*	L-AmB and Flucytosine → Fluconazole and Flucytosine<br><br>EVD	Discontinued<br><br>Started on Ocrelizumab	IRIS followed by recovery
Nel and Hui (2025) [100]	57/F	12 (144)	*NR*	Negative	CNS Cryptococcosis	1.3	667	L-AmB and Flucytosine → Fluconazole	Discontinued	Gradual recovery followed by IRIS then complete recovery
Del Poeta et al. (2022) [28]<br><br>(Aggregate data)	22-67/51.8% F	1 (12)-9 (108)	24 patients with other DMT therapy, 1 with Prednisolone pulse therapy, 1 polymedicated (unspecified)	*NR*	Cryptococcal Meningitis	4 patients 0.500 to <0.800, 17 patients 0.200 to <0.500, 8 patients <0.200	*NR*	31/36 → AmB, Flucytosine, Fluconazole<br><br>5/36 → Unspecified fungal treatment<br><br>Remaining 24 cases with no treatment reported	*NR*	26 Recovered/recovering<br>13 deaths<br>1 deteriorating<br>3 unchanged

Abbreviations: L-AmB, Liposomal Amphotericin B; AmB, Amphotericin B; DMT, disease modifying therapy; ALC, absolute lymphocyte count; HIV, human immunodeficiency virus; MS, multiple sclerosis; CNS, central nervous system; VZV, varicella-zoster virus; IRIS, immune reconstitution inflammatory syndrome; IFN, interferon; VP, ventriculoperitoneal; EVD, external ventricular drain; *NR*, Not reported.

*Fingolimod discontinued 1 month prior to presentation

**Presented 6 months after Fingolimod discontinued

[Table S3] Reported Cases of Other Cryptococcal Infections in Patients Receiving Fingolimod

Study	Age/Sex	Fingolimod Duration in Years (Months)	Prior DMT/Immunosuppression	HIV Status	Diagnosis	ALC at Presentation (cells × 10^9/L)	Absolute CD4+ Count at Presentation (cells/mm^3)	Antifungal Treatment	MS Medication Modification	Outcome
Dahshan et al. (2021) [103]	49/M	9 (108)	*NR*	Negative	Primary Cutaneous Cryptococcosis	0.3	*NR*	Fluconazole	Continued	Complete recovery
Patil et al. (2020) [102]	63/M	7 (84)	High-dose corticosteroids	Negative	Primary Cutaneous Cryptococcosis	*NR*	13	Fluconazole	Discontinued	Complete recovery
Forrestel et al. (2016) [104]	62/F	3 (36)	No recent systemic steroids, otherwise, *NR*	Negative	Primary Cutaneous Cryptococcosis	0.65	56	Fluconazole	Discontinued	Complete recovery
Gibson and Mcgraw (2024) [105]	33/F	5 (60)	*NR*	*NR*	Primary Cutaneous Cryptococcosis	0.22	*NR*	*NR*	*NR*	*NR*
Gencebay et al. (2024) [106]	56/F	5 (60)	*NR*	Negative	Primary Cutaneous Cryptococcosis	1.3	*NR*	Fluconazole	Discontinued<br><br>Started on Dimethyl Fumarate	Complete recovery
Lo and Lee (2020) [101]	52/F	2 (24)	*NR*	Negative	Cutaneous Cryptococcoma	0.7275	91.7	AmB and Flucytosine → Fluconazole with Flucytosine Tumor excised	Discontinued<br><br>Started on Glatiramer Acetate	Complete recovery
Samudralwar (2019) [107]	45/M	3 (36)	Corticosteroids, IFN Beta-1a and 1b	*NR*	Pulmonary Cryptococcosis	0.680	*NR*	Fluconazole	Discontinued<br><br>Started on Glatiramer Acetate (and later Dimethyl Fumarate)	Partial recovery
Calabresi et al. (2014) [108]	*NR*	*NR*	*NR*	*NR*	Asymptomatic Pulmonary Cryptococcosis	*NR*	*NR*	Itraconazole	*NR*	Complete recovery
Harirchian et al. (2020) [29]	43/F	4 (48)	Unspecified MS Medications	*NR*	Necrotizing Cryptococcal Osteomyelitis	1.12	*NR*	Voriconazole	*NR*	Complete recovery
Carpenter (2022) [109]	46/F	11.67 (140)	Methylprednisolone, Glatiramer, Dexamethasone	Negative	Cryptococcal chest wall mass with rib Osteomyelitis	0.3	*NR*	L-AmB and Flucytosine → Fluconazole	Discontinued	Complete recovery

Abbreviations: L-AmB, Liposomal Amphotericin B; AmB, Amphotericin B; DMT, disease modifying therapy; ALC, absolute lymphocyte count; HIV, human immunodeficiency virus; MS, multiple sclerosis; CNS, central nervous system; IFN, interferon; *NR*, Not reported.
